# Deliberative processes in health technology assessment of medicines: the case of Spain

**DOI:** 10.1017/S0266462323000387

**Published:** 2023-07-05

**Authors:** Pilar Pinilla-Dominguez, Jaime Pinilla-Dominguez

**Affiliations:** Department of Quantitative Methods, Management, University of Las Palmas de Gran Canaria, Las Palmas, Spain

**Keywords:** Spain, health technology assessment, health policy, pricing, stakeholder engagement

## Abstract

**Objectives:**

Spain incorporated in 2020 changes in its health technology assessment (HTA), pricing, and reimbursement system for medicines including publishing reports, development of networks of experts, or consultation with stakeholders. Despite these changes, it is unclear how deliberative frameworks are applied and the process has been criticized for not being sufficiently transparent. This study analyses the level of implementation of deliberative processes in HTA for medicines in Spain.

**Methods:**

We review the grey literature and summarize the Spanish HTA, pricing, and reimbursement process of medicines. We apply the deliberative processes for HTA checklist, developed to assess the overall context of the deliberative process, and identify the stakeholders involved and type of involvement following the framework for evidence-informed deliberative processes, a framework for benefit package design that aims to optimize the legitimacy of decision making.

**Results:**

In the Spanish HTA, pricing, and reimbursement process deliberation takes place in order to exchange viewpoints and reach common ground, mainly during the prioritization, assessment, and appraisal steps. It is closed to the public, not clearly summarized in published documents and limited to the Ministry of Health, the regulatory agency, other Ministries, and experts with mostly clinical and/or pharmaceutical background. The views of stakeholders are only represented through consultation. Communication is the most commonly used form of stakeholder engagement.

**Conclusions:**

Despite improvements in transparency of the Spanish HTA process for evaluating medicines, aspects related to stakeholder involvement and implementation of deliberative frameworks need further attention in order to achieve further legitimacy of the process.

## Background

Innovation in health care is coming at a faced pace. Breakthrough health technologies are offering promising benefits to patients and healthcare systems alike. However, the proliferation of such advancements has also contributed to substantial rise in healthcare costs (1). Ensuring that limited healthcare budgets are spent efficiently and in an equitable manner is, therefore, paramount (2). Health technology assessment (HTA) is a tool that contributes to such an objective, and it is defined as “a multidisciplinary process that uses explicit methods to determine the value of a health technology at different points in its lifecycle. The purpose is to inform decision making in order to promote an equitable, efficient, and high-quality health system” (3).

HTA decisions are deemed as some of the most difficult decisions in public health care since they involve the allocation of usually scarce healthcare resources. Therefore, principles such as those known as accountability for reasonableness are due regard in public decision making including HTA (4). These principles include publicity, relevance, appeal, and enforcement. Given the relevance and impact of such decisions, the process should also be fair, transparent, and acceptable to the stakeholders and the wider public. Daniels and van der Wilt (5) note that a fair process involves deliberation about the reasons, evidence, and rationales that are considered relevant to meeting population health needs fairly. The HTA process has different stages where deliberation can take place including horizon scanning, prioritization of topics, provision of scientific advice, scoping, assessment including synthesis of evidence, contextualization of HTA through appraisal, development and communication of the output, and monitoring and evaluation. For deliberation to be applied, there needs to be an exchange of views and perspectives between participants (6). Specifically, in HTA deliberation should include the informed and critical examination of an issue, through the weighing of arguments and evidence to support a decision. Although HTA agencies across the world apply deliberation differently, it is uncommon that the overarching principles for implementing them are transparently and clearly described (6–9).

In the case of Spain, there is evidence that the Spanish society is interested in being informed about medicine and health, and that amongst different science and technology areas, medicine (new diseases, vaccines, etc.) is considered the highest priority area for applied research (10). There is also evidence that the Spanish society considers that the public should be involved in decisions about science and technology that have a direct impact on them, with 29 percent of the respondents confirming so in 2020 (10), indicating the public’s willingness to be involved in health and care decision making.

Spain incorporated in 2020 a series of changes in the HTA and pricing and reimbursement system for medicines (11). This includes publishing reports, development of networks of experts, inclusion of consultation steps, and publishing the process for the evaluation, pricing, and reimbursement of medicines in the Spanish national health system (NHS). Despite these improvements, there is still a perceived lack of transparency in the process (12) and the level as to which deliberative frameworks are applied in this context is unclear.

This study assesses the level of implementation of deliberative processes in HTA for medicines in Spain. Based on these findings, we identify areas for improvements in the process and make actionable recommendations.

## Methods

In order to assess the level of deliberation implemented in each of the steps of the HTA process for evaluating medicines in Spain, we first review the grey literature, specifically the official documents from the Ministry of Health’s Web site, the Spanish Medicines Regulatory Agency (in Spanish, Agencia Española de Medicamentos y Productos Sanitarios [AEMPS]) Web site and legislative documents covering the medicines’ pricing and reimbursement process in Spain (see Supplementary Table 1). We then summarize the process for the HTA evaluation, pricing, and reimbursement of medicines in the Spanish NHS.

Secondly, we apply the deliberative processes for HTA checklist developed by the Health Technology Assessment international (HTAi) and the International Society for Pharmacoeconomics and Outcomes Research (ISPOR) joint task force to assess the overall context of the deliberative process in the Spanish HTA process and to understand the principles underpinning the deliberation (6). The authors note that some of the questions included in the checklist relate to the key features of a deliberative process that should be stated in the terms of reference (see Table 1). In this study we explore these key questions, providing an assessment of the level as to which these features are covered in the official documents in Spain. We also identify the stakeholders involved and type of involvement at each step of the process following the framework for evidence-informed deliberative processes from Oortwijn et al. (8), which provides practical guidance to establish evidence-informed deliberative processes in HTA for health benefit package design based on stakeholder involvement as a way to optimize the legitimacy of the decision-making process. The level of stakeholder involvement is categorized in three levels: participation, consultation, and communication. Participation is the more advanced form of stakeholder involvement and includes deliberation. Consultation is limited to the process for unilaterally collecting feedback from stakeholders, and communication refers to information sharing via different channels.

**Table 1.** Key features of a deliberative process

Phase	Question
The need for a deliberative process	(i) Why deliberate? (ii) What are desired outcomes of the deliberative process? (iii) What is the scope of deliberation?
The preparation for a deliberative process	What are the guiding principles?
Conducting a deliberative process	(i) Who deliberates? (ii) What membership arrangements enable effective deliberation? (iii) How will participants be selected? (iv How are perspectives to be represented? (v) How will participants’ identities be disclosed? (vi) How open should the deliberation be? (vii) What is the type of deliberation needed? (viii) What is the length of deliberation needed? (ix) What are the rules of deliberation? – If deliberation is used to provide an opinion (e.g., advice or recommendation), who has voting rights? – How are criteria made available to guide an exchange of viewpoints? – If deliberation is used to provide an opinion (e.g., advice or recommendation), how is the deliberation to end? (closure)

Based on these analyses, we arrive to actionable recommendations.

Ethical approval was not sought nor required for this review and analysis. All the data used in this article are in the public domain.

## Results

### The Spanish HTA, Pricing, and Reimbursement of Medicines

**Figure 1.**
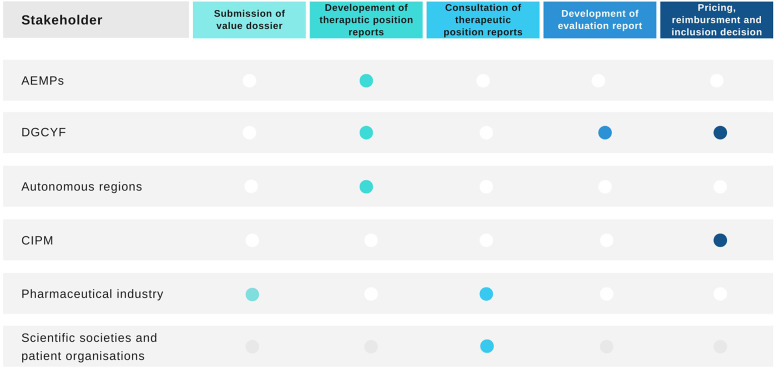
Health technology assessment process in Spain and stakeholders involved. AEMPS, Spanish Medicines Regulatory Agency; CIPM, Drug Pricing Interterritorial Commission; DGCYF, Ministry of Health’s General Directorate for Essential Benefits Package of the NHS and Pharmacy. *Source:* Elaborated by the authors.

The Spanish HTA process, pricing, and reimbursement of medicines is complex and involves several groups of stakeholders (see Figure 1) (13). It has been described as dysfunctional, confusing, and disorganized (14). First, the medicine needs to receive marketing authorization by the European Medicines Agency or the AEMPS in order to be considered for evaluation, pricing, and reimbursement. Once the medicine has marketing authorization, the topics are listed for evaluation and the pharmaceutical company that holds the marketing authorization for the medicine needs to confirm the intent to commercialize the product and apply for pricing and reimbursement in the NHS to the Ministry of Health’s General Directorate for Essential Benefits Package of the NHS and Pharmacy (in Spanish, Dirección General de Cartera Básica de Servicios del Sistema Nacional de Salud y Farmacia [DGCYF]).

All new licensed medicines and new indications listed for evaluation are then prioritized (11). The process of prioritization and the subsequent evaluation is conducted by the DGCYF which collaborates with AEMPS and the different autonomous regions in the form of a public network for the evaluation of medicines named RevalMed to develop a therapeutic positioning report (in Spanish, Informe de Posicionamiento Terapeutico [IPT]) (13). The Permanent Pharmacy Commission of the Ministry of Health is responsible for the governance of this process. They also approve the prioritization of topics for the development of IPTs. However, it is questionable whether these responsibilities are supported by the current legislation (14). The objective of the IPT is to summarize the available clinical and economic evidence on the particular medicine with the aim to identify its appropriate positioning within the pathway of care. It resembles the format of an HTA report. Once a topic has been prioritized, the pharmaceutical company is invited to submit evidence. The scoping process for the IPTs then follows a PICO framework (i.e., specifying the population, intervention, comparators, and outcomes). This is conducted by RevalMed (11). There is no consultation or publication of the scope.

The IPTs are then developed by the RevalMed. The clinical part of the IPT is drafted by the staff from the DGCYF and AEMPS, and the pharmacoeconomic aspects are drafted by the DGCYF. The autonomous regions that participate in the development of the IPTs as part of the RevalMed do so organized in clinical evaluation nodes, based on the therapeutic area under consideration (11).

The first draft of the IPT is subject to consultation with specific stakeholders including scientific societies, patient organizations, and the pharmaceutical industry that holds the marketing authorization of the medicine under evaluation. At this stage, the IPT does not have a specific positioning for the medicine (11;13). The draft IPT is published after the comments from consultation are considered. The process from authorization until this draft publication is expected to take approximately 90 days (15).

Once the IPT has been developed, a different evaluation report is drafted by the DGCYF to inform pricing and reimbursement (13). The pricing and reimbursement recommendation is made by the DGCYF and the Drug Pricing Interterritorial Commission (In Spanish, Comision Interterritorial de Precios de Medicamentos [CIPM]). The members of the CIPM arrive to a conclusion on the price for the medicine and make recommendations on the reimbursement of the technology, either as per the license covered in the marketing authorization, for a subgroup of patients or under specific circumstances. They can also recommend not to fund the medicine (16). The pharmaceutical industry can submit allegations, following publication of the summary conclusions.

The specific positioning of the medicine within the treatment pathway is only proposed by the RevalMed and included in the IPT at the final stage after pricing and reimbursement have been agreed by the DGCYF and the CIPM. The final inclusion/exclusion decision in the NHS is responsibility of the DGCYF (13).

### Deliberative Processes in HTA and Pricing and Reimbursement in Spain

#### Determining the Need for a Deliberative Process

##### Why Deliberate?

The overarching goal of deliberation applied in the overall HTA process in Spain is not specifically stated in any document. Public documents on the HTA process and procedures include aims such as: “To optimize the procedure for the evaluation of medicines in the NHS” and “To gain a bigger compromise and dedication of the participants involved in the evaluation process” (11). The legislation requires that an evaluation takes place before pricing and reimbursement decisions are made. The process defining the pricing and reimbursement decision making provides details on how deliberation takes place, with the aim to improve transparency.

##### What Are the Desired Outcomes of the Deliberative Process and What Is the Scope of Deliberation?

**Figure 2.**
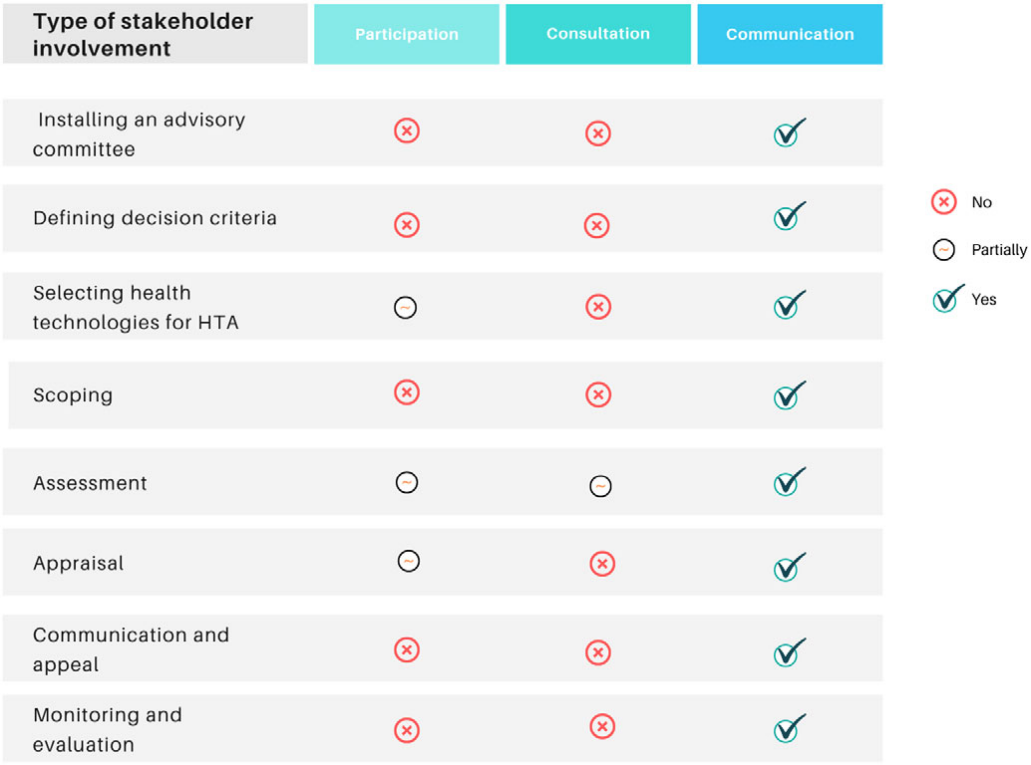
Level of stakeholder involvement in each HTA step. HTA, health technology assessment. *Source:* Elaborated by the authors.

In the Spanish HTA process for the evaluation of medicines, the practices related to deliberation take place in order to exchange viewpoints and reach common ground, mainly during the prioritization of topics and the assessment and appraisal steps. Communication is the most commonly used form of stakeholder engagement (see Figure 2).

During the prioritization, deliberation occurs to discuss and agree on the applicable criteria for each of the medicines considered (11;17). In the assessment step, evidence deliberation occurs between the members of the RevalMed who are experts and specialists. Comments from stakeholders submitted during consultation are considered in the development of the draft IPTs. Finally, during the appraisal step, deliberation takes place to agree the final positioning of the medicine. For the pricing and reimbursement decisions, deliberation takes place to weight up the different criteria for reimbursement, agree on a price and provide a recommendation for reimbursement (see Box 1).

Box 1. Deliberation in the Spanish HTA and pricing and reimbursement process.

*Prioritization of HTA topics*

There is a level of deliberation between the members of the RevalMed, with the possibility of having debates via audioconferencing. Exchange of views between participants also takes place in written format through sharing of comments via a virtual platform (11). There is no consultation with wider stakeholders. The resulting score for each topic is not published, but the list of topics prioritized is published in the AEMPS’s Web site (17).

*Assessment*

Once the IPT has been drafted and consulted upon, comments submitted during consultation are considered by the RevalMed and the experts from the clinical evaluation node, who can arrange further audioconferences for exchanging of views as required. The clinical evaluation and pharmacoeconomic groups of the AEMPS and DGCYF then consider the comments from stakeholders and update the IPT where relevant. After that, the relevant clinical evaluation node considers the updated IPT and they can exchange views between themselves and with the evaluation groups of the AEMPS and DGCYF through audioconferences, if needed. The RevalMed coordination group then has another opportunity to deliberate on the IPT, following all these updates, and will proceed to approve and publish the draft IPT (11).

*Appraisal*

For pricing and reimbursement decisions, the CIPM deliberates using a consensus system. If consensus is not reached, voting is used with a simple majority required to achieve a decision. These meetings can take place in person or in a nonface to face capacity. When the meetings are celebrated in a nonface to face format, electronic means are deemed valid for the deliberation, including email, audioconferences, and videoconferences. Before each meeting, members of the CIPM can submit comments for consideration by other members after receiving the relevant documentation and evaluation report, and before the meeting of the CIPM. Pre-meetings can be arranged where appropriate (16).

AEMPS, Spanish Medicines Regulatory Agency; CIPM, Drug Pricing Interterritorial Commission; DGCYF, Ministry of Health’s General Directorate for Essential Benefits Package of the NHS and Pharmacy; HTA, health technology assessment; IPT, therapeutic positioning report.

#### Preparing for a Deliberative Process

##### What Are the Guiding Principles?

Timeliness, transparency, and efficiency are mentioned as relevant aspects for the HTA process in Spain. The official documents describing the HTA, pricing, and reimbursement process (11;13;16) [NO_PRINTED_FORM] can be understood as terms of reference for the HTA process including deliberative elements (see Supplementary Table 1).

In terms of timeliness, the aim is to develop the IPTs between the time when the decision for marketing authorization is made by the regulators at the commission of human medicines and the final decision at the commission level. This means that some of the steps have short timeframes. This is particularly important for the consultation step with stakeholders. Although the process outlines the timeframe available to submit comments, stakeholders are not notified in advance about when they can expect the IPT to arrive, limiting the ability to plan the provision of comments by the specified timelines.

A trade-off between efficiency and inclusivity can also be perceived. The current process for developing the IPTs aims to optimize the procedures and improve the inclusion and provision of information on elements of scientific and technical nature. The decision-making criteria used in deliberation at different stages of the HTA and pricing and reimbursement process are summarized in Box 2.

Box 2. Criteria used for deliberation in the HTA and pricing and reimbursement process.

*Prioritization of HTA topics*

The prioritization criteria include place in the therapeutic area; potential incremental clinical benefit compared with other alternatives funded in the NHS; similar clinical benefit but better safety profile than other alternatives funded in the NHS; license extension/new indication of an already commercialized and funded treatment; and potential interest for the NHS (11). There is a brief description and scoring for each of them, but no further definitions of the terms used in the description of the criteria are provided (see Supplementary Table 2).

*Assessment*

The factors included in the IPTs are clinical effectiveness and relevance to clinical practice, safety, cost-effectiveness, limitations of the evidence, and budget impact (13). Stakeholders are not involved in the process for defining these criteria.

*Appraisal*

The decision criteria considered for reimbursement are severity; duration and consequences of the condition; specific needs of certain subgroups; social and therapeutic value of the medicines and incremental clinical benefit considering also its cost-effectiveness; rationing of public spending on medicines and budget impact from the perspective of the Spanish NHS; availability of alternative therapeutic options for the condition at the same or inferior price to that of the medicine under consideration; and the level of innovation of the medicine (27). These criteria are not described further.

HTA, health technology assessment; IPT, therapeutic positioning report.

Whereas the process and methodology to develop the IPT are published (18) the assessment process and methods used to develop the evaluation reports are unclear and unpublished. The reports can be requested by the pharmaceutical industry whose medicine is subject to evaluation (13).

Based on the type of participants involved in the HTA process it can be inferred that governance and efficiency play an important role that may hamper the application of a wider and more inclusive deliberative process in Spain, with participation of wider stakeholders being limited.

#### Conducting a Deliberative Process

For prioritization and assessment, deliberation is limited to staff from the evaluation units of DGCYF and AEMPS, and experts nominated by the autonomous regions to take part in the clinical evaluation nodes, mostly experts with clinical and/or pharmaceutical background as part for the RevalMed. The RevalMed nodes are currently comprised of 143 members organized in seven clinical evaluation nodes. The members have the following expertise: pharmacy/pharmacology (69 percent), medicine (28 percent), biology/biotechnology (2 percent), and health economics (1 percent). Each node is led and coled by two different autonomous regions that can have such role for a 2-year term. After the 2-year term, the autonomous region coleading the node becomes the lead coordinator of the specific node (11;13).

No lay members or patient representatives are included in any node. This means that the views of patients and the public are not directly represented in the development of the IPTs. Similarly, the role of health economists is very limited with only two members being part of the nodes. The membership arrangements are not clearly described. There is a closed nomination process conducted by the autonomous regions with final members being appointed based on scientific reasons (undefined) and following declaration of interest and confidentiality agreements (not published). Their identity is not disclosed and they provide their input on a voluntary basis. The number of experts taking part in the clinical evaluation nodes has increased from 120 in November 2020 to 143 people in May 2022 (11;13). This could indicate that membership is permanent or renewable.

The experts from the clinical evaluation nodes can provide their own point of view. Deliberation is closed to the public. Exchange of views can take place in written format through comments shared via a virtual platform and debates are organized through audioconferencing in one or several meetings to allow for discussion between the participants, where required (see Box 1) (11).

During consultation, the views of the patients, the scientific societies, and the pharmaceutical industry is only represented through a passive provision of comments in the IPTs, with no opportunity for active engagement. This limits their ability to deliberate and so, contribute to the process by providing and exchanging views. Although the resulting IPT is published, the individual comments from stakeholders are not published. Their participation is acknowledged.

Although scientific and other factors are described for developing the IPTs (see Box 2) (18), it is unclear how exactly these are considered in the deliberation and in the final proposed positioning of the medicine as the deliberations are not summarized in any document.

For pricing and reimbursement decisions, membership of the CIPM, including their identity is publicly available. This includes representatives from different ministries in Spain and autonomous regions, mostly general directors of pharmacy from the different regions (19). The members from the different autonomous regions are rotated in a periodic basis. The Secretary of State for Health and the DGCYF act as president and vice-president of the CIPM, respectively. The process for the meetings of the CIPM is described and publicly available. The CIPM meets ten times a year, in person or remotely through electronic means (telephone or audiovisual). The members of the CIPM need to declare any conflicts of interest (not published), and they will be excluded from the CIPM if any interest is deemed to be a conflict. The definition of conflict of interest is publicly available (16).

Summaries of each meeting of the CIPM are published with the recommendation and, where the medicine is not recommended, the rationale for exclusion referring to the criteria for reimbursement is provided (20). However, the deliberation is not captured further, there is no document specifying how each of the defined criteria for pricing and reimbursement are considered in the decision-making process or how the IPTs and the evaluation reports influence the appraisal of each of these criteria and the final decision. The evaluation reports and underpinning evidence are not published.

Following pricing and reimbursement decision, the final proposed positioning of the medicine is consolidated by a limited group of participants, these being the evaluators from the AEMPS and DGCYF and the lead and colead of the clinical evaluation node (13). The final IPT is then published in the AEMPS’s Web site (21).

## Discussion

Several studies have assessed the impact of the introduction and evolution of the IPTs in the pricing and reimbursement system in Spain and the efficiency of the process for their development (12;14;22;23). However, they have not conducted an in-depth assessment of the level of implementation of deliberative processes in HTA for medicines in Spain following a best practice framework. This study is the first one attempting this endeavor.

When assessing deliberative processes, it is important to consider political, legislative, and operational factors that influence a particular HTA system (9). Governance, legislative, political, and timeliness factors seem to influence the HTA process for medicines in Spain and thus the level of deliberation applied (14). The fact that health is a devolved matter in Spain explains the emphasis in involving the autonomous regions in the process. The proposed timelines to develop the IPTs, including the timings allocated to each of the steps may implicitly show why a specific type of engagement is chosen. Furthermore, it seems that HTA, in the form of the IPT, is deemed to be an aspect of technical nature, and hence limiting the involvement of wider actors in their development.

Although the mapping of HTA national organizations, programs, and processes in EU and Norway conducted by the European Commission as part of the implementation of the EU HTA legislation notes that Spain considers patient aspects beyond what is reflected in the clinical aspects separately (24), these aspects are not actually separately included in the ITPs. It is also questionable how patient aspects can be assessed without wider patient involvement in the IPTs.

Proper patient involvement requires resources and time to develop communication materials, methods guidance and educational resources (9). Spain will need to improve these aspects by the time the EU HTA legislation enters into force. At the moment, the role of stakeholders like patients or industry is limited to the passive provision of comments on a draft report. Their comments are not published, and it is not possible to ascertain if and how these are considered. This, in turn, makes the consultation process look like a tokenistic exercise rather than a deliberative commitment for inclusivity.

Furthermore, although academics and health economists are part of the RevalMed, their participation is limited to two members. Given the technical nature of the IPTs, and the incorporation of economic evaluation factors, sufficient representation of health economists would enhance the deliberation by the provision of relevant expertise. This could be improved by allowing collaboration between the RevalMed and the Spanish Network for Health Technology Assessment (RedETS), whose remit is to conduct HTA for nonpharmaceutical technologies.

Deliberative processes have been the focus of study in many international HTA discussions, most recently being the chosen topic at the 2020 HTAi Global Policy Forum (9). During this forum, the core principles of deliberative process identified were transparency, inclusivity, and impartiality. While in fact, transparency improvements have recently been made with the publication of several documents in Spain, greater participation of stakeholders, and hence incorporation of inclusivity principles will add legitimacy to the process. Although impartiality seems to be guaranteed by the requirement to declare conflict of interests, the policy and actions taken at a governance level are not published, nor the actual conflicts declared by participants, substantially limiting the application of the principle itself.

The other aspect associated with deliberative processes in HTA that requires further attention in Spain is how the decision-making criteria are ascertained and whether those reflect the values of the Spanish society. Legitimacy of the decision-making process goes beyond information about efficacy, cost-effectiveness, and safety of the technology (5;25). It is also important that the factors guiding decisions reflect societal values. This does not necessarily mean that there needs to be agreement amongst the public. As noted by Daniels et al. (5), people disagree about the trade-offs they are willing to make and the application of common democratic tools to gather public opinion may not necessarily make the process and principles reflect ethical reflections. Therefore, they claim to include moral deliberation, not simply aggregating preferences that people have, but allowing for social learning between stakeholders. Complexity and differing views were also confirmed by findings from the HTAi Global policy forum 2012, where it was recognized that even “the views of a group of informed members of the general public convened specially for the purpose of discussing and advising on citizens’ views of healthcare priorities may vary markedly between members and over time” (26). The factors that guide decision making for the incorporation of medicines in the Spanish NHS as stated in the legislation include aspects such as severity, social, and therapeutic value, or level of innovation (27). How these are defined, interpreted, and considered is however unclear and a matter for further research. Although attempts have been made to study certain trade-offs that some groups of the public have with regards to health systems priorities (28), these have not taken a wider public perspective nor focus specifically on the factors to be considered in HTA decision making. There is no single preferable method to do this (29). Other countries and HTA agencies around the world have attempted to ascertain these preferences and a comparison on public involvement approaches to inform decision-making factors is an area for further research. For example, acknowledging that NICE and its committees “have no particular legitimacy to determine the social values of those served by the NHS,” NICE established its Citizens Council in its begging, to consult a sociodemographic representative sample of the English population on moral and ethical principles that guide the social value judgments applied in their decision making (30). Exercises like this have proven to be complex and costly and have evolved over time. The current form of the NICE Citizens Council is that of the NICE Listens, and it selects a different sample of members of the public to take part in each new topic (31). Despite being difficult, these exercises are proven to be valuable and resonate with the aim to make the process fair, inclusive, transparent, and impartial, in line with the principles for applying deliberative frameworks.

Spain is not an exception in terms of the level of implementation of stakeholder engagement though. Previous studies have shown limited adoption of participatory methods amongst HTA organizations (32). However, agencies around the world seem to be paying more attention to this matter and making efforts to improve their engagement and deliberation methods (12;33). In fact, within the Spanish NHS itself, RedETS have different plans for stakeholder engagement (34;35).

There seems to be momentum in Spain for further improvement in HTA processes following the recent progress made with the new plan for developing IPTs (11). The informative documents published alongside also show that there has been a level of monitoring and evaluation of the HTA process (13). It is important that HTA bodies rigorously document and periodically review their processes, including committee configuration, and assess the impact of any changes over time (9). Organizational and structural changes in Spain may also be warranted to avoid duplication of efforts, improve efficiencies and clarify roles and responsibilities of those involved in the HTA, pricing, and reimbursement process. Therefore, it will be important that Spain monitors the impact of the recent changes, and further investigates the introduction of any further changes as a result. It will also be important that they describe the process, rationale, and reasons for any changes. This will allow further research in studying normative changes applied by HTA agencies.

### Summary of Actionable Recommendations for Improving Deliberative Processes and Implications for Policy Members

- Implementation of deliberative frameworks based on wider stakeholder involvement can improve the transparency, accountability, and legitimacy of the Spanish HTA, pricing, and reimbursement process for medicines.
- Actionable recommendations include:
  - Specify the aims of the deliberation and the principles supporting it.
  - Incorporate deliberation in all HTA stages such as in scoping.
  - Involve wider stakeholders such as academics and lay members in the deliberation at different stages.
  - Open the deliberation to public observance or at least capture the deliberation in the published documents specifying how each factor contributed to the final decision.
  - Add further consultation steps, for example during prioritization and scoping.
  - Make the names, the declaration of interest, and the nomination process of members of the RevalMed public.
  - Publish and retain in the public domain the draft and the final version of the therapeutic positioning reports.
  - Publish the prioritization results and the scope before the evaluation starts, the comments made by the stakeholders during consultation, the evaluation reports, and all the evidence underpinning all the reports.
- In order to address these areas for improvement, it may be necessary to continue evolving the HTA process for medicines, organizational structures, and responsibilities in Spain. Any changes to the process should be monitored and evaluated.

## Conclusions

Although several improvements have been made in terms of transparency of the HTA process for evaluating medicines in Spain in the recent years, aspects related to stakeholder involvement and implementation of deliberative processes need further attention in order to improve the inclusivity, impartiality, and transparency of the process, in line with best practice and enhancing its legitimacy.

## Supplementary material

For supplementary material accompanying this paper visit https://doi.org/10.1017/S0266462323000387. click here to view supplementary material
