# Supplementary material for: Deliberative processes in health technology assessment of medicines: the case of Spain
Source: Int J Technol Assess Health Care. 2023 Jul 5;39(1):e50. doi: 10.1017/S0266462323000387 (PMC11569961; doi:10.1017/S0266462323000387)
Supplement: Supplementary file 1 [file S0266462323000387sup.zip › S0266462323000387sup001.docx]

**Article title:** Deliberative processes in health technology assessment of medicines: the case of Title of Manuscript: DELIBERATIVE PROCESSES IN HEALTH TECHNOLOGY ASSESSMENT OF MEDICINES: THE CASE OF SPAIN

Running Title: Deliberation in Health Technology Assessment in Spain

**Supplementary file 1:** Official documents and reports on the evaluation, pricing and reimbursement of medicines in Spain

Table S1. Official documents and reports on the evaluation, pricing and reimbursement of medicines in Spain

| Name | Weblink and reference | Publication year |
| --- | --- | --- |
| Real Decreto Legislativo 1/2015, de 24 de Julio, Por El Que Se Aprueba El Texto Refundido de La Ley de Garantías y Uso Racional de Los Medicamentos y Productos Sanitarios | [BOE.es - BOE-A-2015-8343 Real Decreto Legislativo 1/2015, de 24 de julio, por el que se aprueba el texto refundido de la Ley de garantías y uso racional de los medicamentos y productos sanitarios.](https://www.boe.es/buscar/doc.php?id=BOE-A-2015-8343#:~:text=A%2D2015%2D8343-,Real%20Decreto%20Legislativo%201%2F2015%2C%20de%2024%20de%20julio%2C,los%20medicamentos%20y%20productos%20sanitarios.) | 2015 |
| Informes de posicionamiento terapeutico | <https://www.aemps.gob.es/medicamentos-de-uso-humano/informes-de-posicionamiento-terapeutico/> | 2013 – 2022 |
| Reuniones del GC de la REvalMed SNS | <https://www.aemps.gob.es/medicamentos-de-uso-humano/informes-de-posicionamiento-terapeutico/reuniones-del-gc-de-la-revalmed-sns/> | 2022 |
| Reglamento interno de la comisión interministerial de precios de los medicamentos (CIMP) | <https://www.sanidad.gob.es/profesionales/farmacia/pdf/REGLAMENTO_CIMP_30_09_2019.pdf> | 2019 |
| Comisión interministerial de precios de medicamentos y productos sanitarios | <https://www.sanidad.gob.es/profesionales/farmacia/CIPMyPS.htm> | 2017 – 2022 |
| Preguntas y respuestas frecuentes sobre el sistema de información para determinar el valor terapéutico en la práctica clínica real de los medicamentos de alto impacto sanitario y económico en el sistema nacional de salud | [20191021.VALTERMED Preguntas y Respuestas.DGCYF (sanidad.gob.es)](https://www.sanidad.gob.es/en/profesionales/farmacia/valtermed/docs/VALTERMED_Preguntas_y_Respuestas.pdf) | 2019 |
| Plan para la consolidación de los informes de posicionamiento terapeutico de los medicamentos en el Sistema Nacional de Salud | [20200708.Plan_de_accion_para_la_consolidacion_de_los_IPT.actCPF8Julio.pdf (sanidad.gob.es)](https://www.sanidad.gob.es/profesionales/farmacia/IPT/docs/20200708.Plan_de_accion_para_la_consolidacion_de_los_IPT.actCPF8Julio.pdf) | 2020 |
| Procedimiento Normalizado De Trabajo De Evaluación Clínica , Evaluación Económica Y Posicionamiento terapeutico para la redaccion de informes de posicionamiento terapeutico de medicamentos en el sistema nacional de salud | <https://www.sanidad.gob.es/gl/profesionales/farmacia/IPT/docs/20200708.PNT_elaboracion_IPT_CPF8Julio.pdf> | 2020 |
| Documento informativo sobre la financiación y fijación de precio de lo medicamentos en España | <https://www.sanidad.gob.es/profesionales/farmacia/pdf/20220526_Doc_Infor_Financiacion_Med_Esp.pdf> | 2022 |
