# Supplementary material for: Deliberative processes in health technology assessment of medicines: the case of Spain
Source: Int J Technol Assess Health Care. 2023 Jul 5;39(1):e50. doi: 10.1017/S0266462323000387 (PMC11569961; doi:10.1017/S0266462323000387)
Supplement: Supplementary file 1 [file S0266462323000387sup.zip › S0266462323000387sup002.docx]

**Title of Manuscript:** DELIBERATIVE PROCESSES IN HEALTH TECHNOLOGY ASSESSMENT OF MEDICINES: THE CASE OF SPAIN

**Running Title:** Deliberation in Health Technology Assessment in Spain

**Supplementary file 2:** Prioritisation criteria for developing therapeutic positioning reports In Spain

Table S2. Prioritisation criteria for developing therapeutic positioning reports

| Criterion | Description and score |
| --- | --- |
| Place in the therapeutic area | Meeting an unmet therapeutic area in a condition based on its level severity in scale of 0 (not meeting the criterion), 5 (meeting the criterion in a non-severe condition) and 10 (meeting the criterion in a severe condition) |
| Potential incremental clinical benefit compared with other alternatives funded in the NHS | Yes=10, Only in a subgroup=5, No=0 |
| Similar clinical benefit but better safety profile than other alternatives funded in the NHS | Yes=5, No=0 |
| Licence extension/new indication of an already commercialised and funded treatment | Yes=10, No=0 |
| Potential interest for the NHS | 0 (no relevance) to 20 (high relevance)  Factors considered include: no unmet need but new management of prevalent condition or with particular clinical interest; no unmet need but technologies currently available to manage the condition have a high budget impact; the technology has conditional marketing authorisation; the technology is licensed for a high impact indication). |
